# Bent pinkies: Quantifying fifth finger clinodactyly in a sample of U.S. adults

**DOI:** 10.1371/journal.pone.0271734

**Published:** 2022-07-20

**Authors:** Myoung Keun Lee, Zelda T. Dahl, Joel Anderton, Jennifer L. Maurer, Mary L. Marazita, John R. Shaffer, Seth M. Weinberg

**Affiliations:** 1 Department of Oral and Craniofacial Sciences, Center for Craniofacial and Dental Genetics, University of Pittsburgh, Pittsburgh, PA, United States of America; 2 Department of Human Genetics, University of Pittsburgh, Pittsburgh, PA, United States of America; Ohio State University, UNITED STATES

## Abstract

Mild curvature of the fifth finger (or clinodactyly) is a relatively common trait. While severe forms can cause functional impairment and are a feature of certain congenital syndromes, mild clinodactyly is considered a minor morphological variant. Despite exhibiting continuous variation, clinodactyly is rarely treated as a quantitative trait. Consequently, the degree of fifth finger curvature in the general population and the factors that impact this curvature are not well understood. In the present study, we measured fifth finger curvature in a sample of 1,295 U.S. adults and investigated the role of sex, age and body size. We found that clinodactyly exhibited a non-normal distribution. All participants displayed some degree of curvature, but it tended to be slight with an overall mean of 3.68 degrees (median: 3.58 degrees). In only 0.8% of cases did the curvature exceed the nominal 10-degree threshold for clinically meaningful clinodactyly. We did not find statically significant sex differences. Further, there was no meaningful relationship with height and only a weak positive relationship with age. We found that clinodactyly showed asymmetry; the curvature was greater on the left than on the right fifth finger (p < 2.2e^-16^), but this was not influenced by sex, age, or height. These results suggest the possibility that the kind of ubiquitous mild clinodactyly observed in the general population may be etiologically distinct from more rare and severe forms of the condition.

## Introduction

Clinodactyly can be broadly defined as curvature of the finger(s) occurring distal to the metacarpophalangeal joint(s). While the curvature can involve any finger and the deviation can be toward the ulnar or radial side of the forelimb, the most common form of clinodactyly involves a radial curvature of the fifth finger [1]. While fifth finger clinodactyly is a well known feature of several syndromes (e.g. Down syndrome), some degree of curvature is also common in the general population [2]. Estimates of incidence in the general population vary widely. For example, De Marinis and De Marinis [3] determined that 15.2% of boys and 8% of girls exhibited fifth finger clinodactyly in a sample of 1387 children 5–12 years old. In contrast, Marden et al. [4] reported an incidence of only 1% in a sample of 4,412 newborns, when no other malformations were present. Age at evaluation may be one factor underlying such discrepancies, as some reports suggest that clinodactyly tends to worsen during maturation [5]. Another factor driving variation in reported incidence may be how the trait is defined. Clinodactyly is a continuous morphological trait. When defined as a qualitative (present/absent) or semi-quantitative (mild/moderate/severe) character state, the threshold used can impact estimates of incidence [6]. Many sources suggest a 10-degree deviation as the threshold for “clinically” meaningful clinodactyly [7], but there is no universally agreed upon standard and in many cases the evaluation is visual and subjective. One consequence of defining clinodactyly in this manner is that a large portion of the population where only a slight degree of finger curvature is present will be excluded. Thus, while clinically significant clinodactyly (however defined) may be relatively rare in the general population, clinodactyly falling within the boundaries of normal-range variation is likely to be much more common.

Because there are few if any functional consequences, the type of mild clinodactyly typically observed in the general population has received little attention. Investigating the full range of phenotypic variation and the factors that influence digit curvature may help resolve conflicting reports about the effects of sex and age on clinodactyly severity. The purpose of this study is to define the degree of fifth finger curvature in a sample of U.S. adults. Because we are interested in the full range of variation, we do not attempt to define a threshold, but rather treat clinodactyly as a quantitative trait. We test whether biological sex and age impact the degree of curvature and evaluate the correspondence between the curvature of the right and left fifth fingers.

## Materials and methods

The individuals comprising the study sample (n = 1295) were recruited from the Western Pennsylvania region as part of two large epidemiological studies focusing on oral health and nonsyndromic oral clefts, respectively. These studies were carried out to investigate genetic and environmental risk factors impacting these oral traits and conditions. As part of these studies, several phenotypic assessments were performed, including hand scans (described in more detail below). The study sample included individuals originally recruited due to their oral/facial conditions or as population-based controls. A total of 18 participants had a nonsyndromic (isolated) cleft of the lip and/or palate. Participants with visible hand deformities or injuries were excluded. Any participants with a known syndrome were also excluded, as clinodactyly is known be a feature of some syndromes. No other exclusion criteria were applied. Participants included in the present study were all adults, ranging in age from 18.2–86.8 years at the time of examination (mean age 37.1 years). The ratio of self-identified males to females was 268(m):1027(f), reflecting the fact that one of the two parent studies recruited only adult females. The self-identified racial and ethnic background of participants was mostly White and non-Hispanic (n = 1180; 91.1%). The next largest group was Asian and non-Hispanic (n = 46; 3.6%), followed by Black and non-Hispanic (n = 15; 1.6%). One participant identified as Native American. The remaining participants were either unknown or reported mixed ancestry (n = 53; 4.1%). The racial and ethnic categories used were required by the funding agency supporting the work. All participants provided their written informed consent before study activities commenced. All studies were approved by the University of Pittsburgh’s Human Research Protection Office: protocols STUDY19080127 and STUDY19080178.

**Fig 1:**
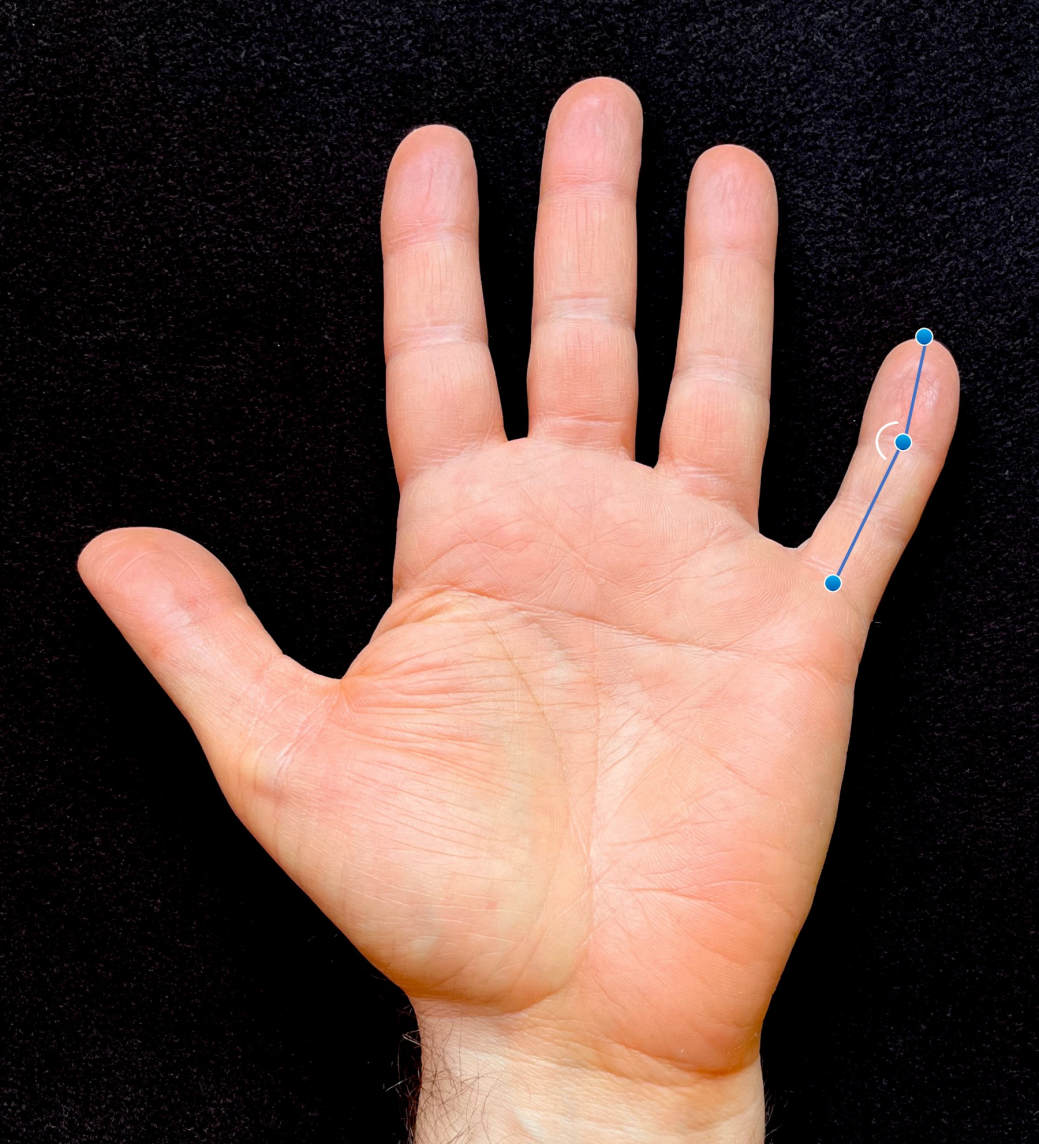
Image showing landmarks used to quantify fifth finger curvature. Landmarks (proximal to distal) include the midpoint of the metacarpophalangeal crease, the distal interphalangeal joint, and the distal margin of the fingertip. The inner angle formed by these points was used to measure the degree of curvature.

As part of broader phenotypic assessment, high-resolution digital hand scans were collected on each participant. Participants were asked to place their hands palm down on a large format flat-bed scanner (Epson GT-20000, Nagano, Japan). Participants were instructed to place their hands on the glass with light pressure and their fingers extended and separated in a natural, unstrained posture. The digital scans were imported into the program tpsDIG2 (http://sbmorphometrics.org/index.html) and landmarks were placed on the fifth finger at the midpoint of the metacarpophalangeal crease, the distal interphalangeal joint, and the distal margin of the fingertip (Fig 1). A prior study of landmark-based finger dimensions obtained from digital scans have shown very low error from repeated measures, with intraclass correlation coefficients exceeding 0.97 [8]. Using x,y coordinates from the collected landmarks, the angle of the distal interphalangeal joint (between the middle and distal phalanges) was calculated to capture the degree of fifth finger radial curvature. In 25 instances only the left or right measure was captured due to problems with the scan or the participant’s hand posture.

Statistics describing the mean, spread and distribution of fifth finger curvature were generated. We report the number of individuals exceeding the threshold for clinical clinodactyly, defined here as a curvature exceeding 10 degrees. Comparison of mean curvature by sex was carried out using a two-sample t-test (or the non-parametric equivalent). The relationship between age/height and curvature was assessed with linear regression. The Pearson’s correlation coefficient was calculated to assess the relationship between left and right figure curvature and an intraclass correlation coefficient (two-way mixed effects, absolute agreement, single rater) was calculated to determine the degree of closeness between the paired measurements. All statistical tests were performed in the R statistical computing environment.

## Results

**Fig 2:**
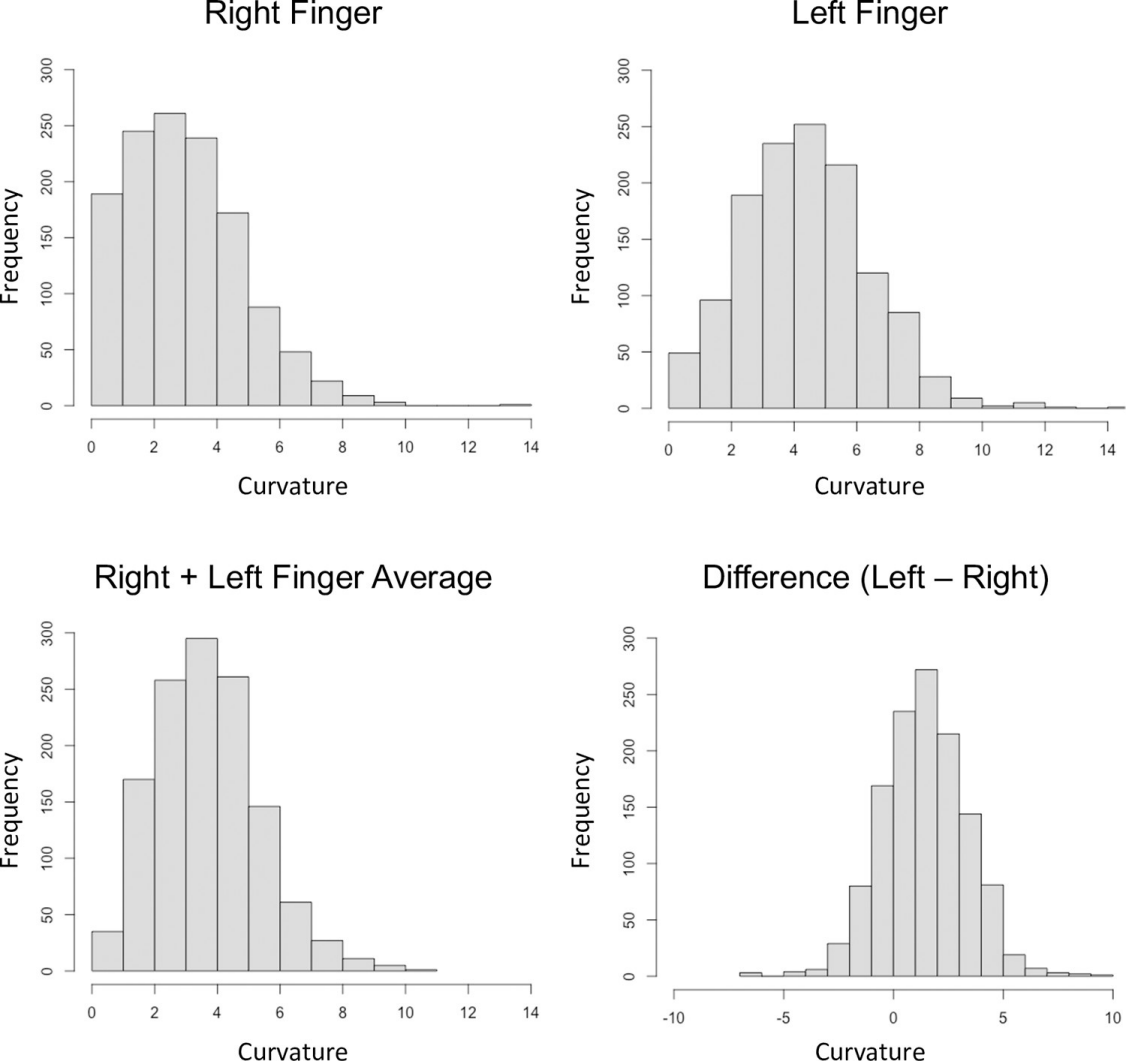
Histograms showing frequency distributions for fifth finger curvature and left-right curvature asymmetry.

**Fig 3:**
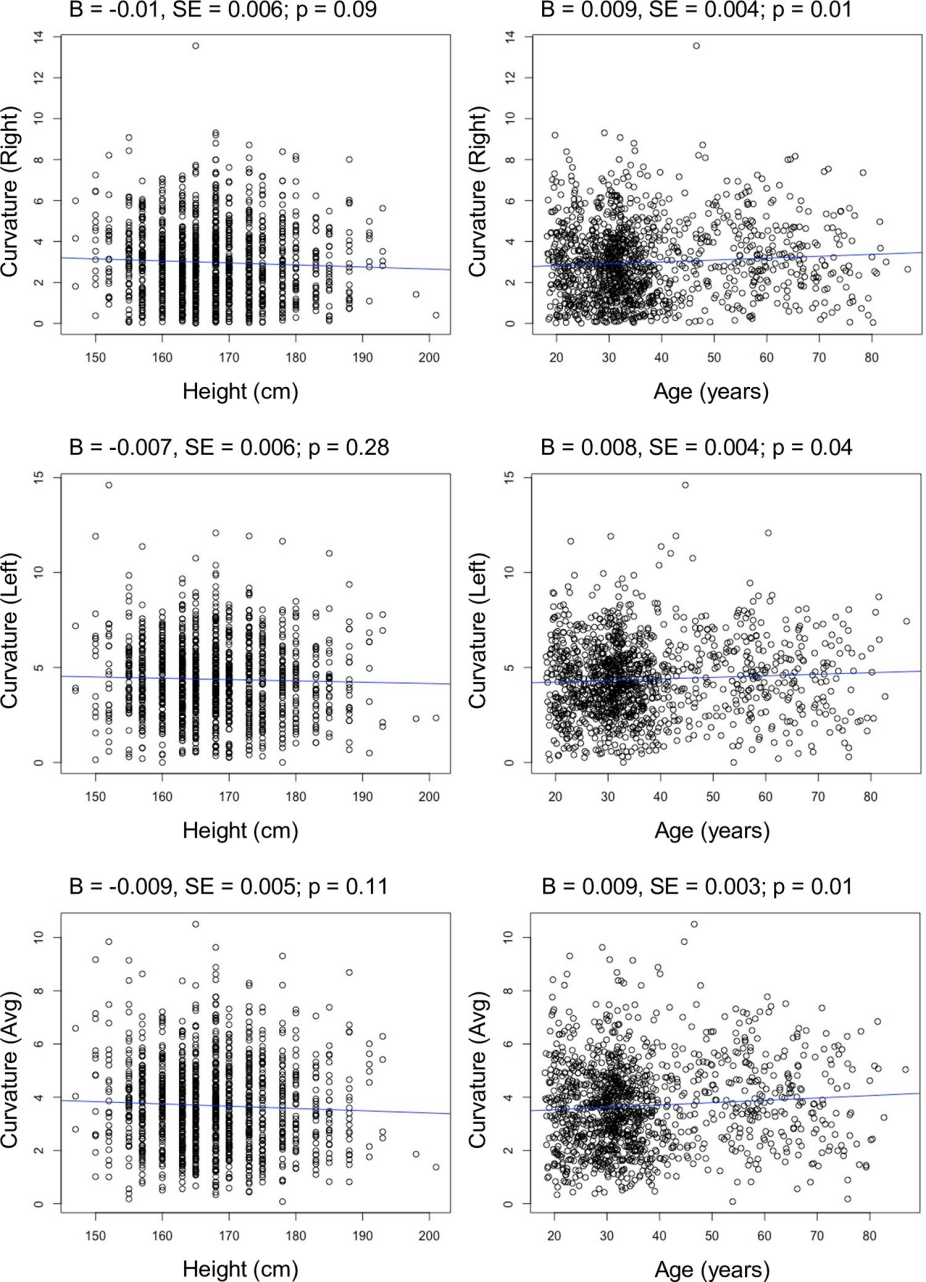
Regression results showing the relationship between height and age with fifth finger curvature. Regression statistics are included at the top edge of each chart: B = unstandardized regression coefficient; SE = standard error of B; p = p-value from test of whether the observed regression coefficient is different from zero.

Basic descriptive statistics (mean, median, sd, range) for fifth finger clinodactyly are provided in Table 1. Clinodactyly did not exhibit a normal distribution; the shape was unimodal but skewed to the right (Fig 2). We found that the average curvature was small at just 2.98 degrees (median: 2.70) for the right finger and 4.38 degrees (median: 4.29) for the left. Only 10 individuals exceeded the nominal clinical threshold of 10 degrees, with the highest recorded value at 14.6 degrees. These 10 individuals did not exhibit any other notable characteristics in terms of their demographic profile. No individuals had a curvature greater than 10-degree on the both the left and right fingers. Based on these data, we observed clinically relevant clinodactyly in only 0.8% of our adult cohort (10/1295), and these were all on the mild end of the spectrum. Non-parametric t-tests (Wilcoxon rank sum) showed no difference in the degree of clinodactyly between males and females: right hand (p = 0.36), left hand (p = 0.82), and combined (p = 0.56). Clinodactyly measures showed no meaningful relationship with height and a weak positive relationship with age (Fig 3). Clinodactyly was greater on the left than on the right fifth finger (Wilcoxon sign rank test; p < 2.2e^-16^), but this difference was not influenced by sex (p = 0.65), age (p = 0.95), or height (p = 0.61). The Pearson correlation showed a moderate relationship between left and right clinodactyly measures (r = 0.49). However, the intraclass correlation showed that the correspondence or agreement between left and right values was poor (r = 0.39). The raw data supporting these analyses is available in S1 File.

**Table 1:** Descriptive statistics for fifth finger clinodactyly.

Variable	Group	n	Mean	sd	Median	Range
Right finger curvature	Male	262	2.89	1.79	2.70	0.10–8.39
	Female	1015	3.00	1.84	2.83	0.01–13.56
	Combined	1277	2.98	1.83	2.80	
Left finger curvature	Male	268	4.37	2.11	4.33	0.004–12.09
	Female	1020	4.38	1.97	4.27	0.002–14.61
	Combined	1288	4.38	2.00	4.29	
Average finger curvature (L,R)	Male	262	3.63	1.66	3.46	0.08–9.31
	Female	1008	3.69	1.64	3.59	0.18–10.50
	Combined	1270	3.68	1.65	3.58	
Difference (Left-Right)	Male	262	1.48	2.00	1.38	-7.00–8.62
	Female	1008	1.38	1.90	1.36	-6.11–9.53
	Combined	1270	1.40	1.92	1.37	

All units are in degrees

## Discussion

In this study, we measured the degree of fifth finger curvature (clinodactyly) present in a sample of U.S. adults. In general, we found that some degree of curvature was ubiquitous, but it was mild in most individuals with our averages falling well below the 10-degree clinical threshold typically used to determine clinodactyly. The 0.8% incidence figure for clinically meaningful clinodactyly in our cohort is in alignment with the 1% figure reported by Marden et al. [4] but differs from the higher incidence estimates reported by others [6, 9, 10]. This may have to do with differences in how the trait was defined or measured or the specific population used in these earlier studies. The main purpose of this study, however, was not to classify individuals, but rather to use a quantitative approach to capture the spectrum of fifth figure curvature present in a reasonably representative population and to probe some of the factors that may drive that variation.

To that end, we examined the effects of self-reported sex, age and height on fifth finger clinodactyly. None of these factors had a substantial impact. The few prior studies that have looked explicitly at sex effects show inconsistent results. Bell [11] and Dutta [12] found that males and females had roughly equal rates of fifth finger clinodactyly. In contrast, De Marinis and De Marinis [3] found that boys had almost twice the rate of clinodactyly as girls in their sample of children. Roche [10] and Skvarilová and Smahel [13] reported a similar sex bias. Interestingly, in the De Marinis and De Marinis [3] study, the sex difference went away when considering only “mild” forms of clinodactyly, which is most similar to phenotype included in the present analysis. This suggests that how the trait is defined may be important and that the sex bias may only be pronounced at the more severe end of the phenotypic spectrum.

We found that age showed a weak positive relationship with degree of curvature. Several clinical reviews on clinodactyly report that the condition gets progressively worse with age resulting from pathological changes at the affected growth plate [5, 7]. However, this claim has rarely been tested. Roche [10] found no age effect in a longitudinal sample of children with down syndrome. More recently, Perrone et al. [6] reported that the rate of clinodactyly *declined* in older children in their cross-sectional Brazilian cohort. These studies, however, are simply looking at the frequency of clinodactyly (defined as a binary character state) in children at different age points. As such, they may not be sensitive enough to capture changes in severity over time. The slight increase in curvature with age that we observed in our adult sample would likely evade any clinical detection. Its cause is also unclear; because our study is limited to adults, it is unlikely to be related to continued changes at the growth plate. Thus, the impact of age on clinodactyly and the pathological processes involved remain unresolved. We found the standing height had no effect. We are not aware of any prior reports that examined the effects of body size on clinodactyly.

When clinodactyly is present, it is often described as presenting bilaterally [1, 3, 12, 13]. De Marinis and De Marinis [3] describe instances of unilateral clinodactyly, but their data suggest the condition is as very rare compared to bilateral presentation. Moreover, when clinodactyly was present unilaterally, it was more common on the right hand. Our findings disagree with these prior reports. We found that fifth finger curvature measures showed little agreement between the left and right sides and that the curvature was significantly greater on the left hand–the opposite of De Marinis and De Marinis [3]. This suggests that–at least at the mild end of the phenotypic spectrum–clinodactyly does not reliably present as a bilateral trait. The possible cause of the observed left bias in our data is not clear.

Overall, our results show that fifth finger curvature (measured as a quantitative trait) in a contemporary U.S. adult sample is both ubiquitous and mild, with almost all individuals falling below even the most liberal clinical clinodactyly threshold. We observed no or very weak effects of both demographic (sex and age) and anthropometric (height) factors. Our sample was almost entirely comprised individuals of European ancestry (self-identified), and thus we were limited in our inability to investigate the role of ancestral background on curvature. A small number of participants had a nonsyndromic orofacial cleft. This subset of individuals did not show any major deviations from the larger cohort in terms of their curvature values and deleting this subset had a negligible impact on the mean curvature for the sample. There is also no prior evidence of an association between clinodactyly and nonsyndromic clefting. Thus, there is no indication that the inclusion of this small number of individuals had an undue influence on our results. We also noted a lack of correspondence in the degree of curvature between the left and right hands. As we have discussed, many of our results disagree with the prior literature. Some of our results may be related to the fact that we focused exclusively on adults and treated clinodactyly as a quantitative continuous trait. These features of our study design made comparison with the existing literature a challenge. An alternative possibility is that, compared to the more severe and clinically relevant forms of clinodactyly typically covered in the literature, the type of mild clinodactyly reported here and present in the general population may have a distinct etiology and therefore exhibit distinct epidemiologic patterns. One possible way to confirm this would be to carry out a radiological examination of a series of both mild cases and more severe cases, which may reveal divergent underlying anatomical causes. Another would be to compare patterns familial transmission. Clinical clinodactyly has been considered an autosomal dominant trait with reduced penetrance for over 70 years [11, 12, 14]. It is unclear whether mild clinodactyly also follows this same inheritance pattern or behaves more like an additive polygenic trait, akin to most continuous morphological features. A modern genetic analysis of finger clinodactyly would help clarify the situation.

## Supporting information

S1 File Complete individual-level data used in the analysis. (XLSX)
